# Synchronous Intercostal Nerve Schwannomas: A Rare Cause of Chest Wall Pain

**DOI:** 10.7759/cureus.78725

**Published:** 2025-02-08

**Authors:** Joana Martins, Ana B Seixas, Mickael Bartikian, Erica Marto, Rafael Roque, Nuno Simas

**Affiliations:** 1 Department of Neurosurgery, Unidade Local de Saude (ULS) Santa Maria, Lisbon, PRT; 2 Department of Anatomy, Faculty of Medicine, University of Lisbon, Lisbon, PRT; 3 Department of Neurosurgery, Unidade Local de Saude (ULS) Lisboa Ocidental, Lisbon, PRT; 4 Department of Orthopedics, Santo André Hospital, Leiria, PRT; 5 Department of Neurology, Unidade Local de Saude (ULS) Santa Maria, Lisbon, PRT; 6 Department of Neuropathology, Unidade Local de Saude (ULS) Santa Maria, Lisbon, PRT

**Keywords:** chest wall pain, encapsulated tumor, intercostal nerve schwannoma, microsurgery, radiculopathy

## Abstract

Schwannomas are nerve sheath tumors arising from Schwann cells. Schwannomas of the intercostal nerve are extremely rare, with limited published literature. These tumors are generally regarded as benign asymptomatic. When symptomatic, they manifest with radiculopathy or chest pain. Surgery is considered when the pain does not respond to medication. Complete resection has a low risk of recurrence or malignant transformation. Herein, we report a pathologically confirmed case of synchronous intercostal nerve schwannoma diagnosed with chest wall pain.

A 38-year-old woman with a breast cancer history treated with neoadjuvant chemotherapy, followed by radical mastectomy and radiotherapy, presented with a three-year history of dorsal pain radiating to the right costal grid (D6 radiculopathy). MRI showed an encapsulated tumor close to the right D6-D7 intervertebral foramen with bone remodeling and a smaller encapsulated tumor close to the costotransverse joint of the right sixth rib. After three years of unsuccessful treatment with pain medication, the patient underwent tumor resection. The right sixth costotransverse joint was exposed with the identification of the smallest tumor. Then, we exposed the right sixth costovertebral joint, and a solid tumor was identified next to the intervertebral foramen. Tumor debulking was carried out with an ultrasonic aspirator.

Postoperatively, the patient experienced significant pain relief, and there were no neurological defects. The neuropathology analysis revealed synchronous intercostal nerve schwannomas. Microsurgical intervention for the management of intercostal nerve neuropathy resulting from dorsal schwannomas has demonstrated a good outcome for the patient.

## Introduction

Schwannomas are a type of peripheral nerve sheath tumor (PNST) arising from differentiated Schwann cells. Although they can occur at any age, they are more common between the ages of 20 and 50 years [1].

Schwannomas of the intercostal nerve are extremely rare, and there is little published literature on them. They most commonly occur in spinal nerve roots. Neurofibromas, the other type of benign PNST, are usually identified in superficial nerves [2].

These tumors affect patients of different ethnicities equally, with no sex predilection. Most cases are sporadic tumors (90%), and the remaining 10% occur in neurofibromatosis type 2 (3%), schwannomatosis (2%), or meningiomatosis with or without neurofibromatosis type 2 (5%) [3,4].

Radiologically, they are well-marginated soft-tissue masses with contrast enhancement. They may contain areas of fat or cystic degeneration, which are spontaneously hyperintense on T2-weighted images and hypointense on T1 [5].

Most benign tumors of the chest wall, including schwannomas, present as slow-growing, painless, palpable masses and are discovered incidentally during imaging of the thorax for other reasons [2,3].

When symptomatic, patients often complain of thoracic continuous belt-like pain, sometimes experienced as shooting pain or chest pain, and difficulty breathing, without any history of trauma. The first line of treatment in those cases is painkillers for neuropathic pain. If symptoms cannot be adequately controlled, surgery is proposed. Most schwannomas of the intercostal nerve manifesting as chest pain are benign tumors [4,5].

Malignant peripheral nerve sheath tumors (MPNSTs) usually show compression or destruction of adjacent structures and pleural abnormalities, suggesting malignancy. Some MRI features are useful in diagnosing MPNSTs, and if a tumor has two or more features, it should be considered highly suspicious for malignancy and proposed for surgery. Those features include mass dimensions larger than 5 cm, the presence of a peripheral enhanced pattern, the presence of perilesional edema, and the presence of intratumoral cystic changes [4,5]. In such cases, localized tumor removal is not enough, and resection of the chest wall is necessary to prevent local recurrence [3,6].

If the tumor is histologically benign, follow-up is unnecessary because the long-term prognosis is very good [3]. We present a case of synchronous intercostal nerve schwannomas along one intercostal nerve, a rare cause of chest wall pain.

## Case presentation

A 38-year-old woman with a history of breast cancer treated with neoadjuvant chemotherapy followed by radical mastectomy and radiotherapy in 2021 presented with dorsal back pain and radiating numbness to the right rib cage (D6-D7 radiculopathy). Considering the history of oncological disease and the absence of trauma, an MRI of the dorsal spine and thorax was performed.

Imaging findings

**Figure 1:**
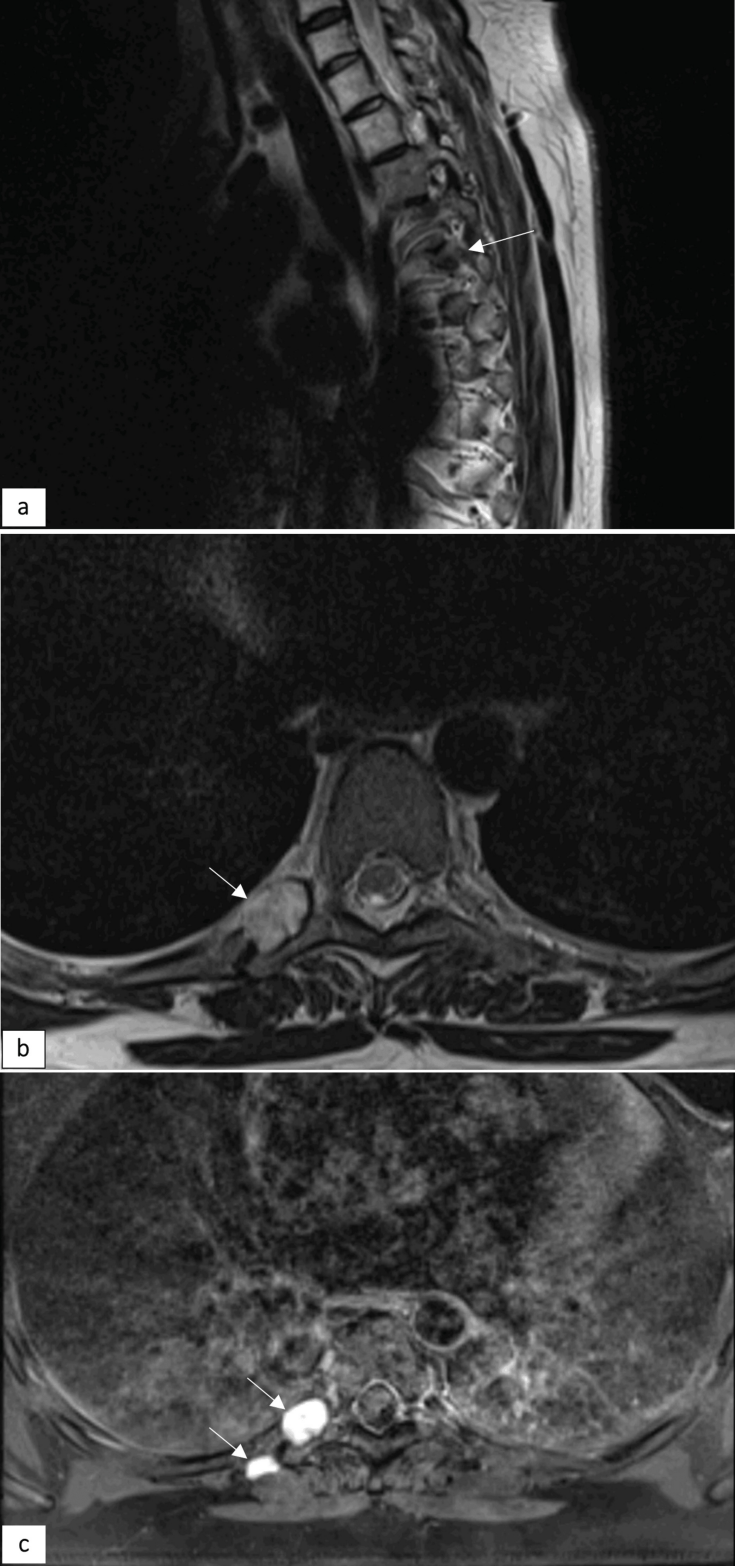
Sagittal (a) and axial (b) T2WI images showing hyperintense masses (arrows) and bone remodeling of the ipsilateral intervertebral foramen. The lesions have a low signal on T1WI. Axial T1W TSE (c) showing homogeneous bright enhancement of the masses (arrows) T2WI: T1-weighted imaging; T1W TSE: T1-weighted turbo spin echo

Dorsal spine MRI showed an encapsulated tumor close to the right D6-D7 intervertebral foramen, with remodeling of the transverse process and the head of the right sixth rib (tumor A), as well as a smaller encapsulated tumor adjacent to the costotransverse joint of the right sixth rib (tumor B) (Figure 1).

Thorax MRI did not show any metastasis or abnormalities besides these two neoplasms. Furthermore, a positron-emission tomography (PET) scan and a rib biopsy were performed, which ruled out metastasis from breast cancer.

Management

After three years of treatment with neuropathic pain medication (pregabalin and amitriptyline), achieving only a partial response, the patient underwent tumor resection.

**Figure 2:**
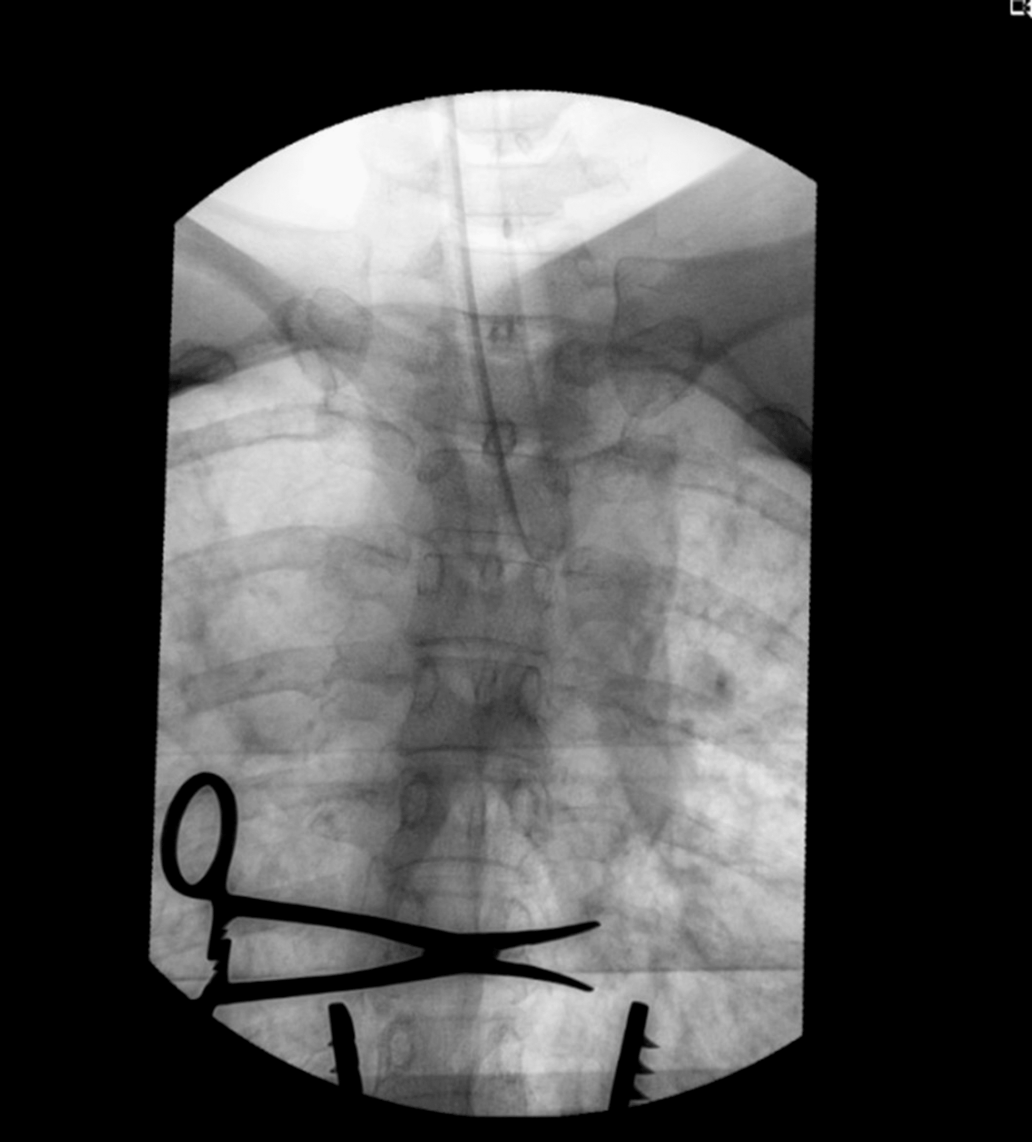
Fluoroscopy of the dorsal spine and rib cage. The forceps indicates the D6-D7 intercostal level on the right side

A 7-cm longitudinal midline incision was made in the dorsal region, followed by unilateral exposure right to the dorsal spine and confirmation of the D6-D7 level using fluoroscopy (Figure 2).

**Figure 3:**
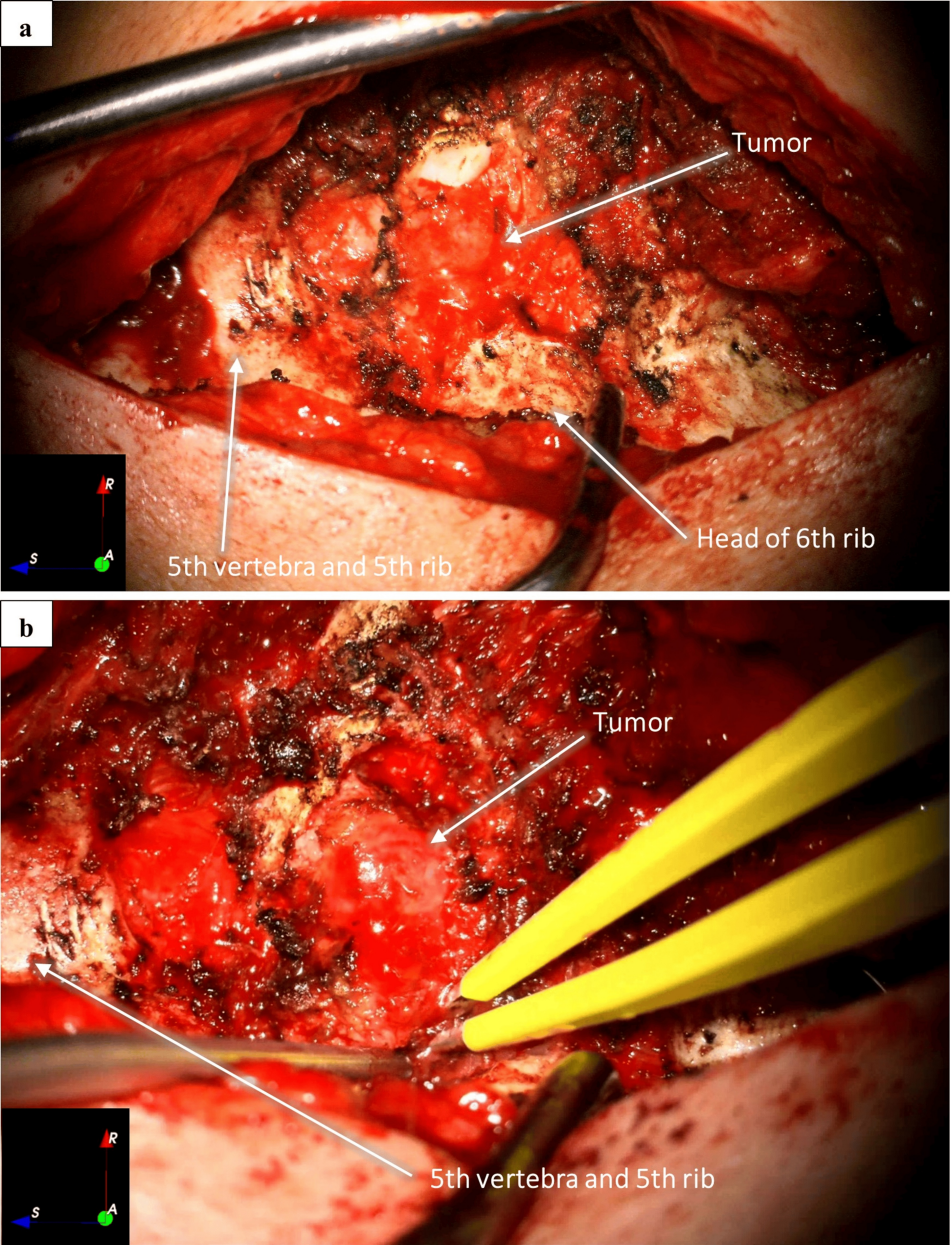
Right lateral exposure of the dorsal spine and ribs, after removing right transverse process of D6 with tumor exposure and dissection. Overall (a) and magnified (b) views

The right sixth costotransverse joint was exposed with the identification of the smallest tumor, which was macroscopically removed. We removed the right transverse process of D6, and a solid tumor was identified next to the intervertebral foramen, along with rib remodeling underneath (Figure 3).

**Figure 4:**
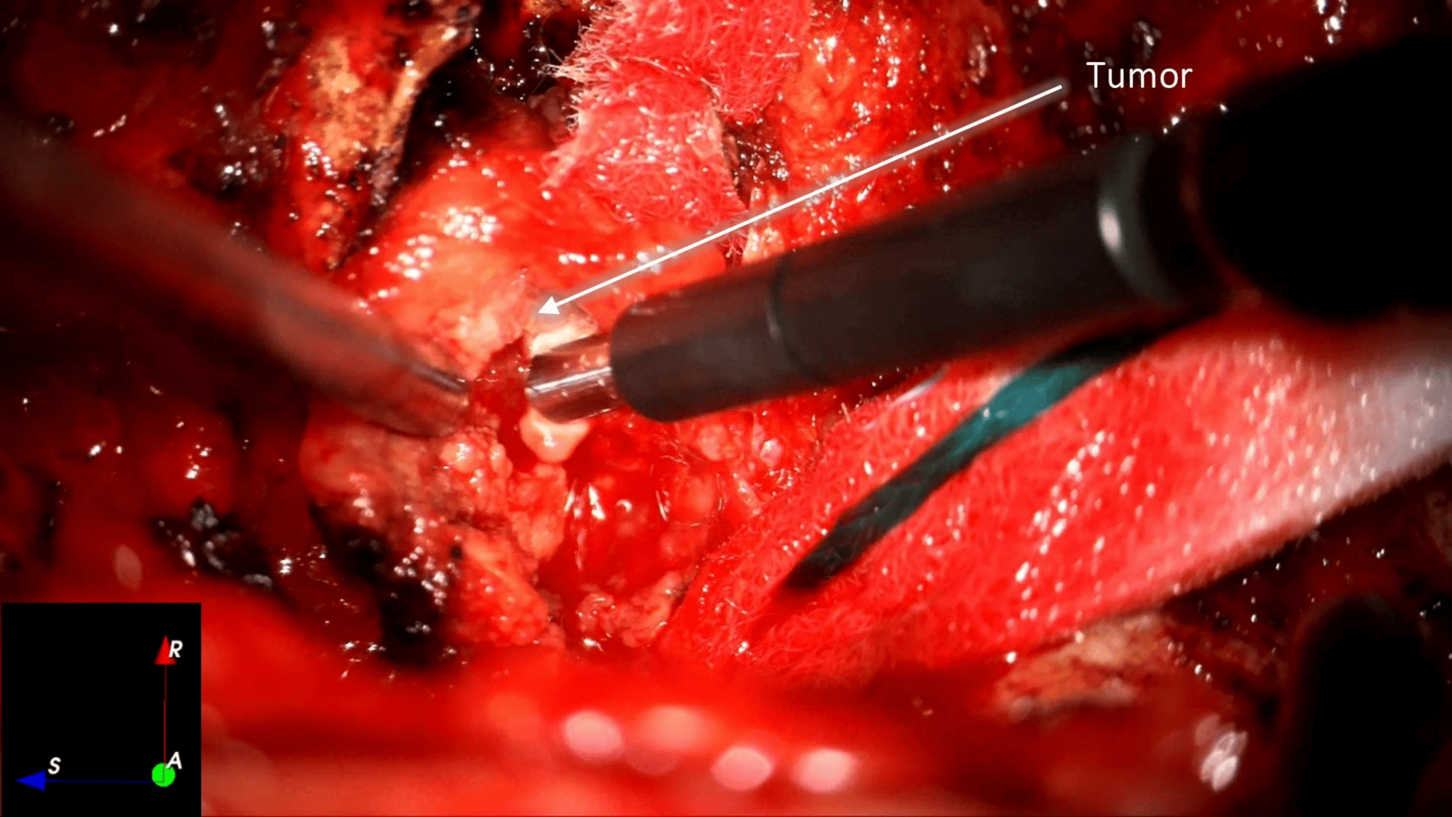
Tumor debulking with ultrasonic aspiration

**Figure 5:**
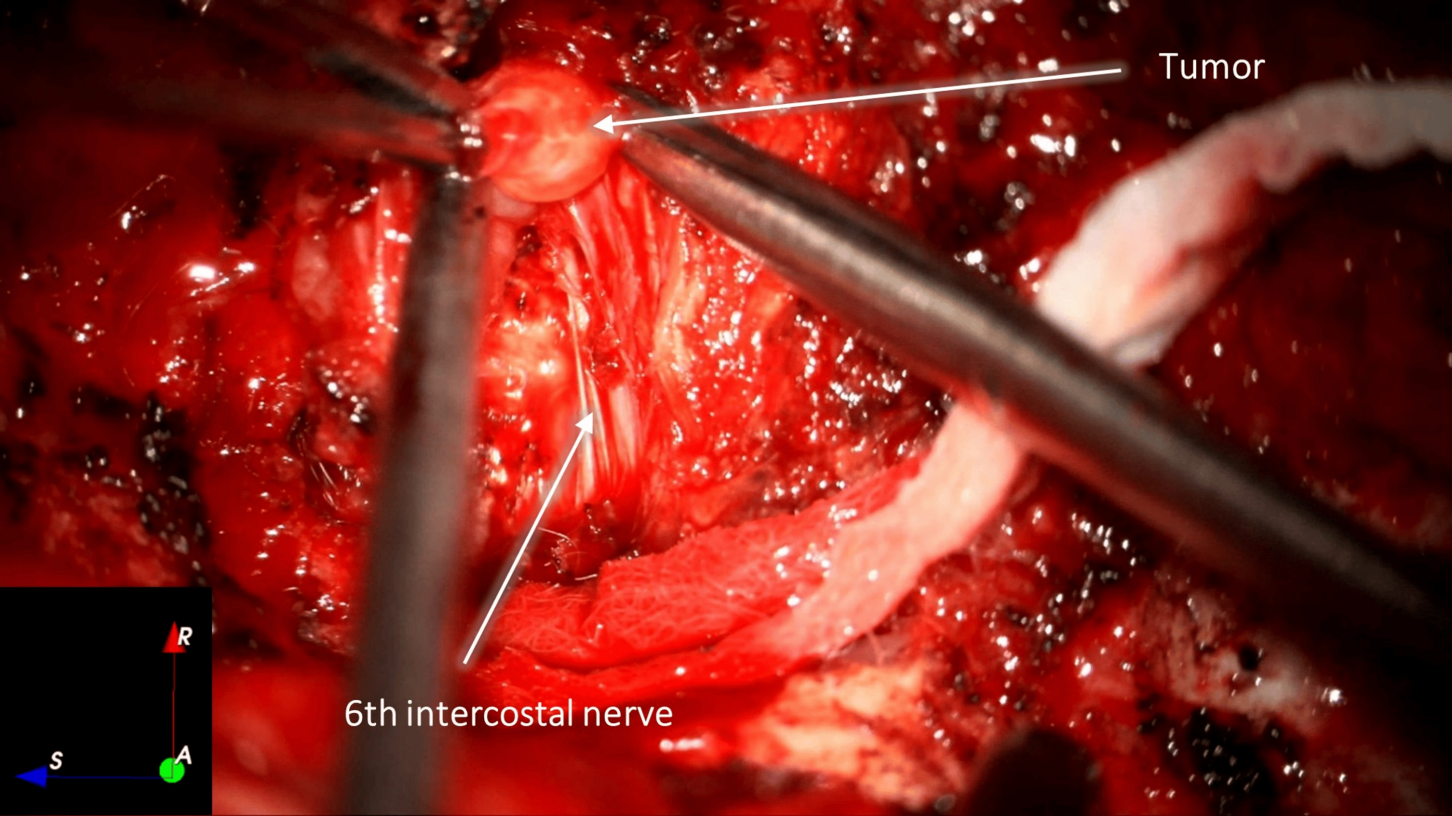
Debulking and sharp dissection of the tumor

**Figure 6:**
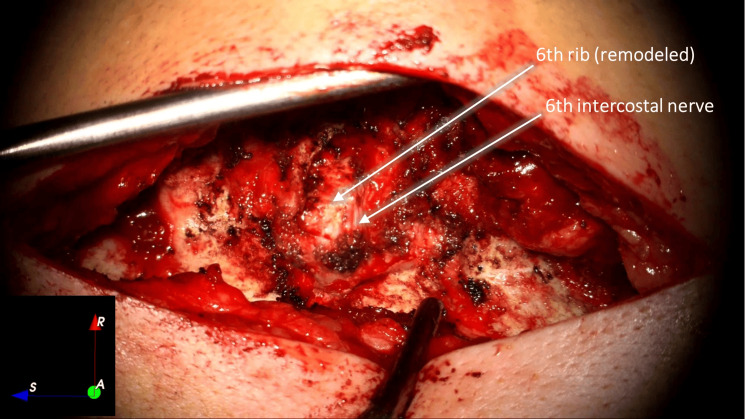
Final exposure after tumor removal and remodeled rib head

Tumor debulking was performed microsurgically using an ultrasonic aspirator (Sonopet®, Stryker, Kalamazoo, MI) and sharp instrument dissection. The tumor and its capsule were macroscopically removed from the right sixth intercostal nerve (Figures 4-6).

There were no postoperative complications, and the patient was discharged one day after surgery. She reported immediate relief from the numbing pain on the right side of the rib cage.

Histopathology

**Figure 7:**
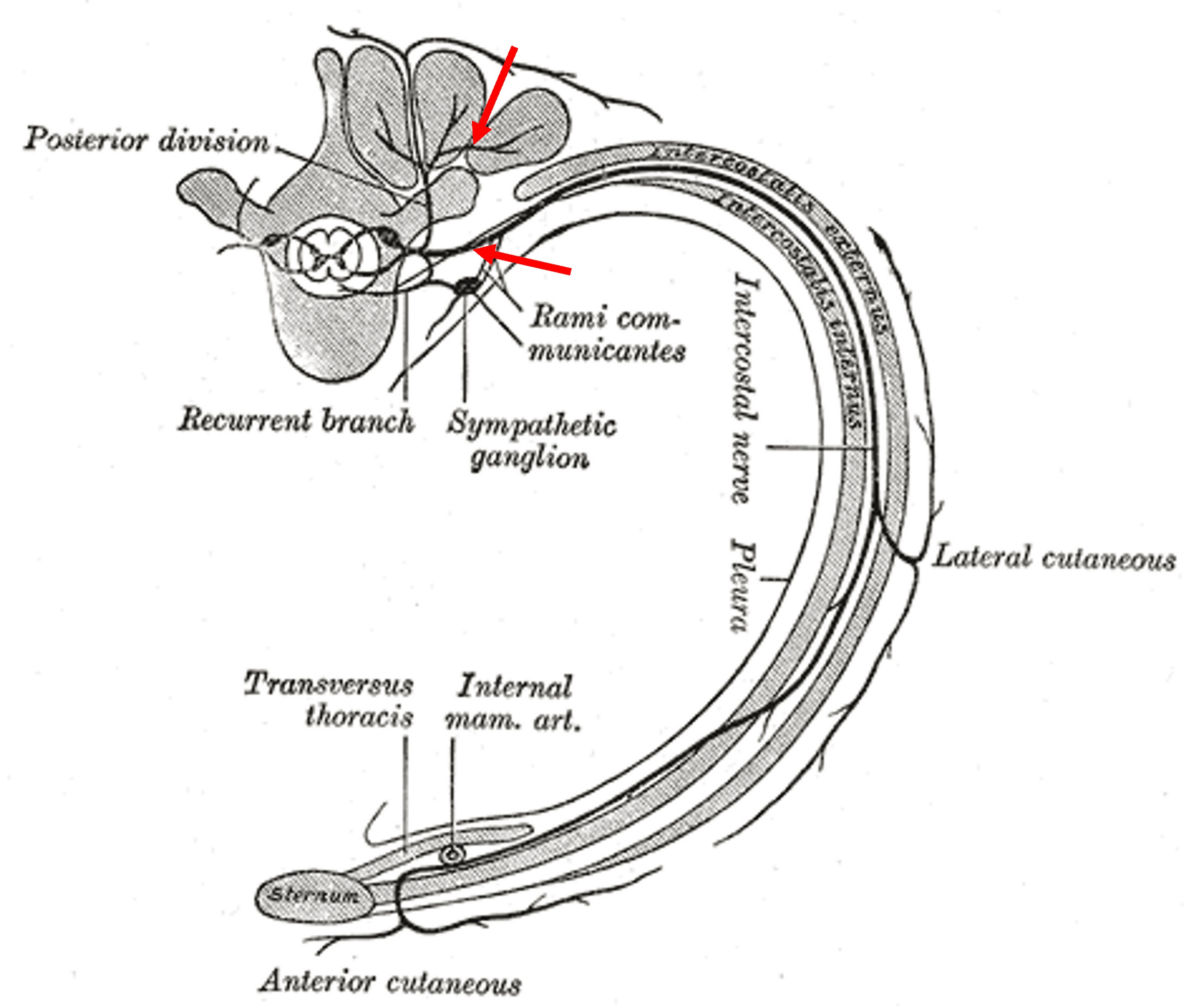
Illustration of intercostal nerve and rib cage with the schwannomas’ locations (red arrows). Source: Adapted from [7], which is currently in the public domain. Published under Creative Commons CC0 License (https://creativecommons.org/publicdomain/zero/1.0/)

The neuropathological findings revealed synchronous intercostal nerve schwannomas, which were located along the dorsal (arrowhead) and ventral (arrow) rami of the nerve, as shown in Figure 7.

**Figure 8:**
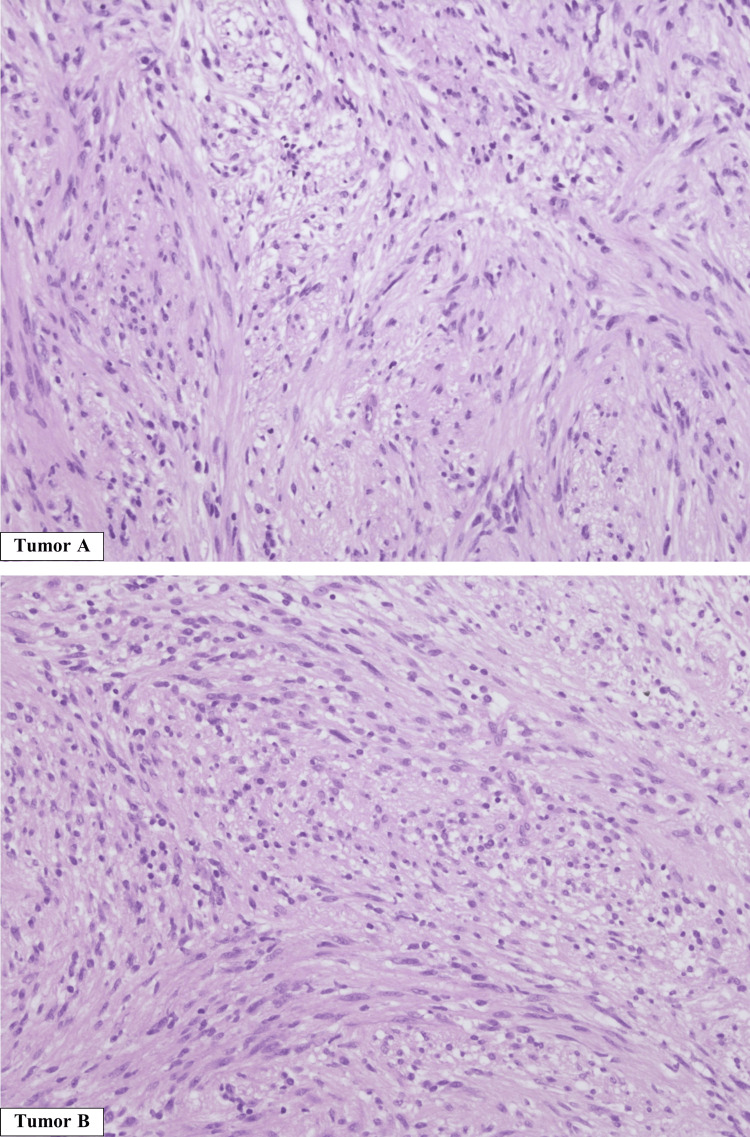
Tumor A (hematoxylin and eosin, 200×) and tumor B (hematoxylin and eosin, 200×) were both encapsulated tumors, with a biphasic morphology of compact elongated cells, with some foci of more loosely arranged tissue. No atypical histological features were seen. Both tumors had diffuse staining for S100 and SOX10 and were negative for EMA and SSTR2A EMA: epithelial membrane antigen; SSTR2A: somatostatin receptor 2A

The histopathological analysis showed two well-demarcated lesions, both with a biphasic morphology of compact elongated cells and some foci of more loosely arranged tissue. The tumors were S100 positive on immunohistochemistry (Figure 8).

Follow-up

**Figure 9:**
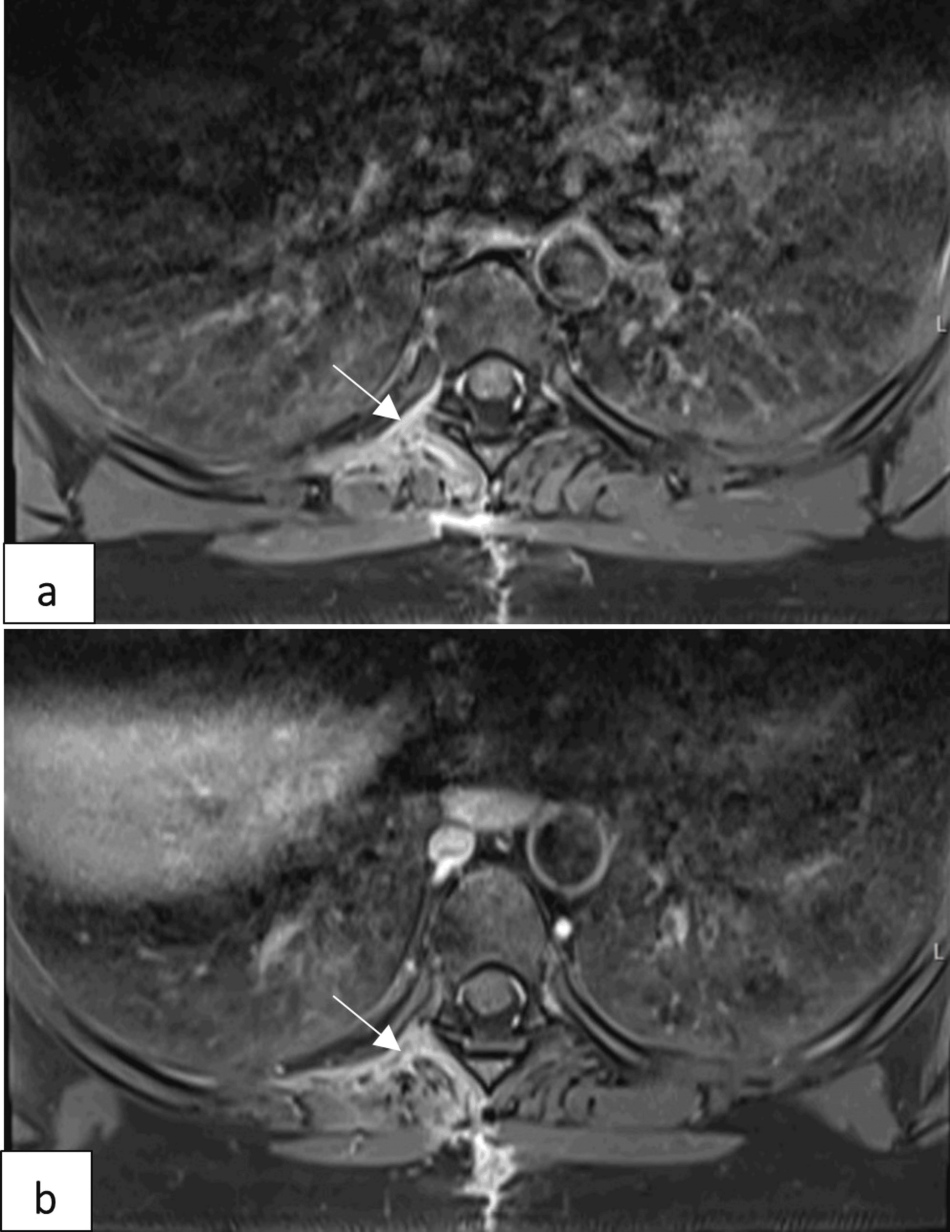
Axial T1 gadolinium-enhanced MRI images four months after surgery, from cranial (a) to caudal (b) at the level of the vertebral bodies D6 and D7, showing fibrosis along the incision and surgical site (arrows), with no apparent masses inside

At four months postoperative follow-up, the patient remained without complaints of radiating or numbing pain, and the MRI scan from that period is shown in Figure 9.

## Discussion

In their classic form, schwannomas are benign, nonrecurring tumors that occur most commonly in adults between the second and fifth decades of life. They are evenly distributed between genders [8].

Schwannomas grow from Schwann cells, which involve peripheral nerves. Thus, they are more likely to be found in the head (e.g., vestibular schwannomas) and neck region, flexor surfaces of the extremities, posterior mediastinum, and retroperitoneum [8].

Schwannomas of the chest wall are uncommon and reportedly manifest as a painless mass, being a late-diagnosed cause of chest wall pain since its rarity. However, in our patient, the tumors were diagnosed earlier than what is described in other case reports, where the diagnosis time reached up to seven years from the onset of symptoms [1,8]. A likely explanation of our rapid diagnosis is the prior history of breast cancer, which motivated more imaging exams in the early stage of the evaluation.

Similarly, our patient’s MRI scan revealed an ovoid lesion along the right sixth rib with bony remodeling, hypointense on T1WI, spontaneously hyperintense on T2WI, and displaying homogeneous bright enhancement on T1Gad, consistent with findings previously reported by other authors [3,4].

Nevertheless, in our patient, two synchronous lesions were present in the same intercostal nerve, which is not very frequent according to the literature (less than 12 cases have been reported to date) [9].

According to several authors, microsurgical resection is the standard treatment for peripheral nerve sheath spinal tumors [3,8]. However, some patients have poor performance status, advanced age, or many comorbidities, making them poor surgical candidates. In those scenarios, other treatment options, such as stereotactic body radiotherapy (SBRT), can be discussed.

Regarding this case, the patient was young and had no other comorbidities other than previous history of breast cancer. In this case, it was important to have a histopathological confirmation of the diagnosis of schwannoma, even though the patient had already done a PET scan and reported no abnormality. Nevertheless, it is advisable to have a histopathologic diagnosis before SBRT. Since the two lesions were close to each other and had feasible anatomical access without breaching the thoracic cavity, the excision of both tumors could be promptly achieved instead of performing a needle biopsy followed by SBRT.

When comparing tumor resection and SBRT in PNSTs, there are no published studies specifically regarding intercostal nerve schwannomas, although more solid evidence has been reported for vestibular schwannomas and intradural extramedullary tumors (e.g., meningiomas, schwannomas, or neurofibromas). Regarding the latter, some studies on radiotherapy have shown radiographic response in 28%-39% of the lesions, and large series report local control in 83%-100%, but no study features median follow-up beyond 37 months [10-12].

Another advantage, besides the instantaneous resolution of pain after surgery, is that wide surgical excision may also prevent malignant transformation (which has also been reported in these tumors) [8].

Our patient’s complaints were resolved immediately after surgery, concordantly with the literature [3]. In our patient, there were no findings of malignancy, such as tumor size >5 cm^2^, bone involvement, or muscle disruption. The histology did not reveal malignancy features such as a high cellularity rate or atypia, which is associated with a good prognosis and a low risk of recurrence [3].

## Conclusions

Intercostal nerve schwannomas are a rare cause of chest wall pain. We described a case of synchronous intercostal nerve schwannomas. There are very few published similar cases in the literature. When symptomatic and unresponsive to pain medication, surgical excision is the gold standard treatment. The surgical removal can be performed microsurgically, resulting in a good outcome for the patient and no associated complications, as we have seen in this case.
